# Supplementary material for: Condensation of Benzyl Carbamate with Glyoxal in Polar Protic and Aprotic Solvents
Source: Molecules. 2023 Nov 17;28(22):7648. doi: 10.3390/molecules28227648 (PMC10675631; doi:10.3390/molecules28227648)
Supplement: Supplementary file 1 [file molecules-28-07648-s001.zip › molecules-2727035-supplementary.pdf]

Supplementary Materials

Condensation of Benzyl Carbamate with Glyoxal in Polar Protic and Aprotic Solvents

Artyom E. Paromov

Laboratory for Chemistry of Nitrogen Compounds, Institute for Problems of Chemical and Energetic Technologies, Siberian Branch of the Russian Academy of Sciences (IPCET SB RAS), Biysk 659322, Russia; nitrochemistry@mail.ru

Table of contents

^{1}H and ^{13}C NMR of compound **2** S2

^{1}H and ^{13}C NMR of compound **3** S4

^{1}H and ^{13}C NMR of compound **4** S6

^{1}H and ^{13}C NMR of compound **5** S8

^{1}H and ^{13}C NMR of compound **7** S10

^{1}H and ^{13}C NMR of compound **8** S13

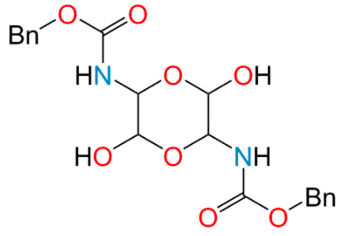

N,N′-bis(carbobenzoxy)-3,6-diamino-1,4-dioxane-2,5-diol (**2**)

^{1}H (400 MHz) NMR (DMSO-D6, 24°C)

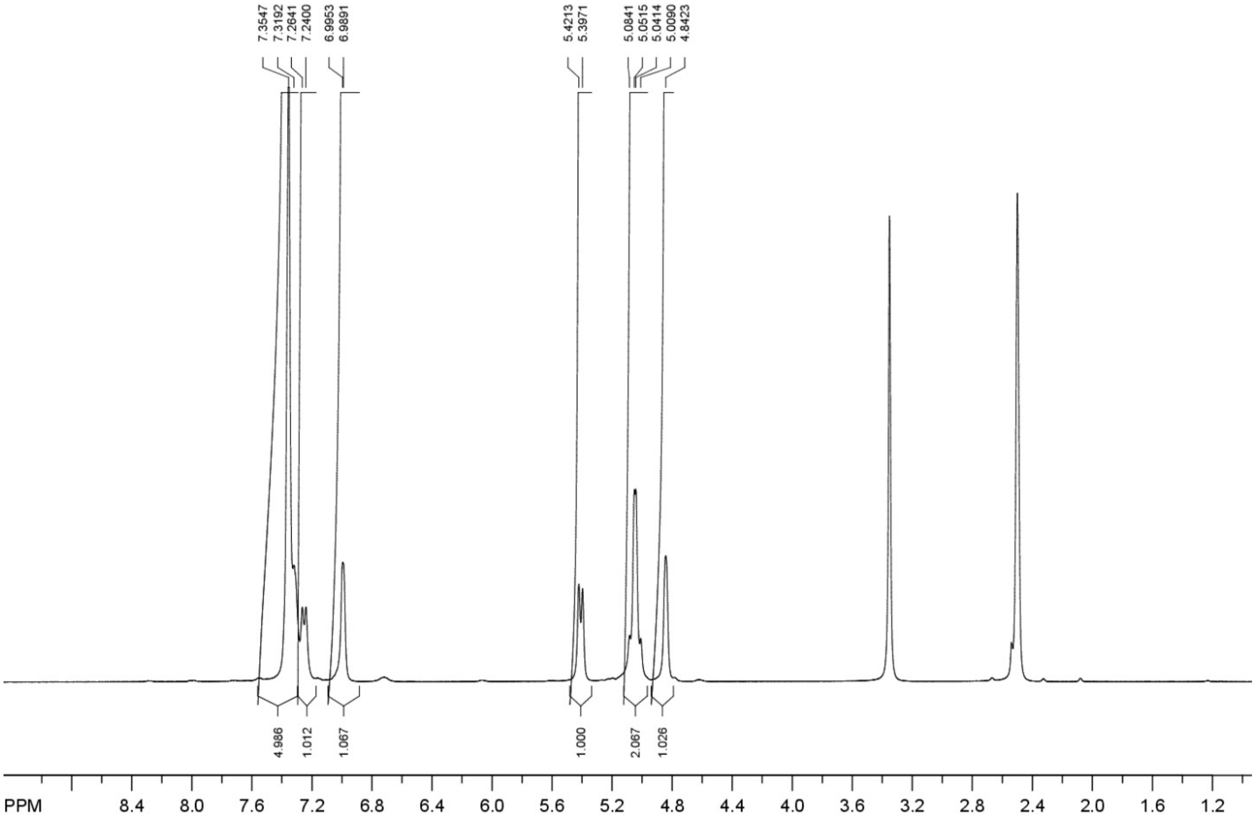

Figure S1. ^{1}H NMR (DMSO-D6) spectrum of compound **2**.

^{13}C (100 MHz) NMR (DMSO-D6, 24°C)

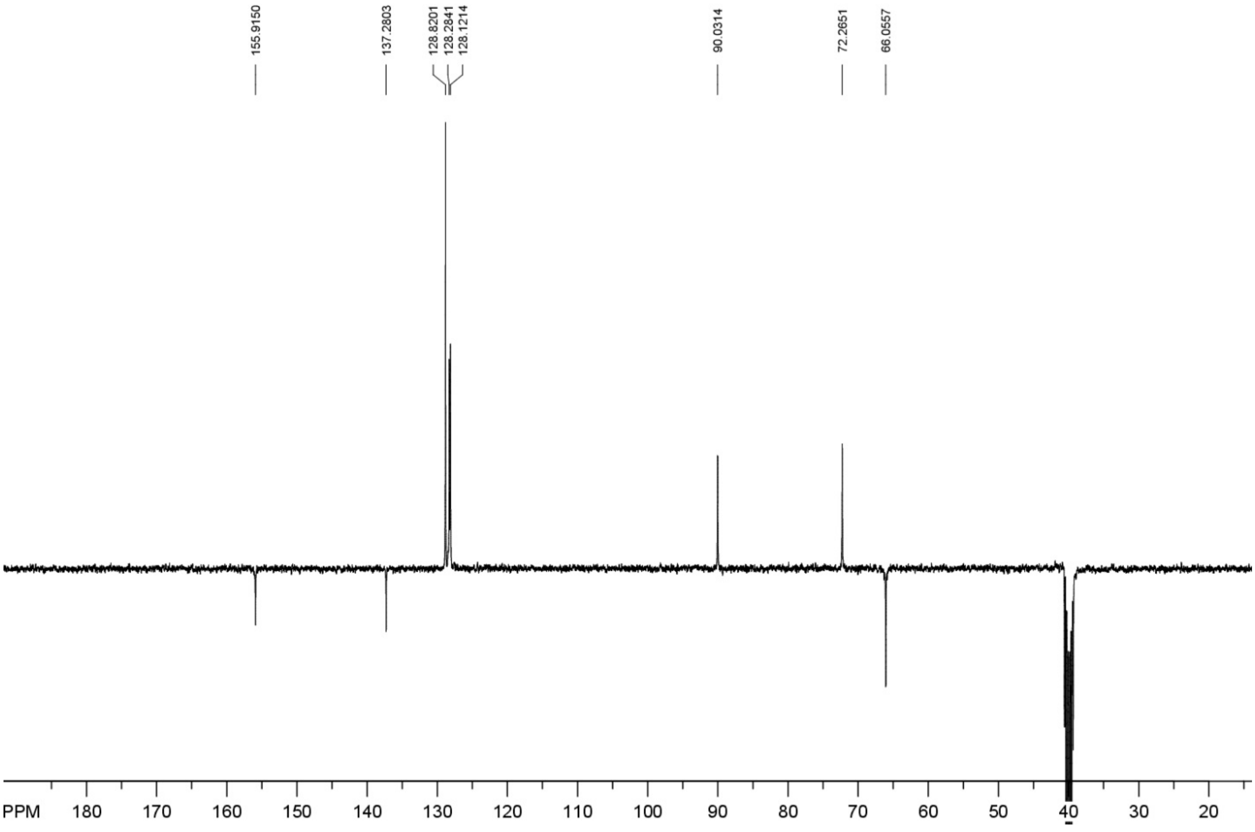

Figure S2. ^{13}C NMR (DMSO-D6) spectrum of compound **2**.

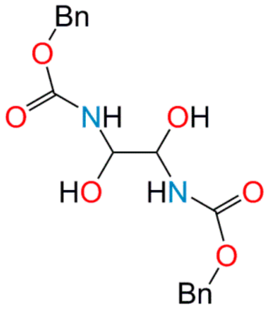

N,N′-bis(carbobenzoxy)ethan-1,2-diol (**3**)

^{1}H (400 MHz) NMR (DMSO-D6, 24°C)

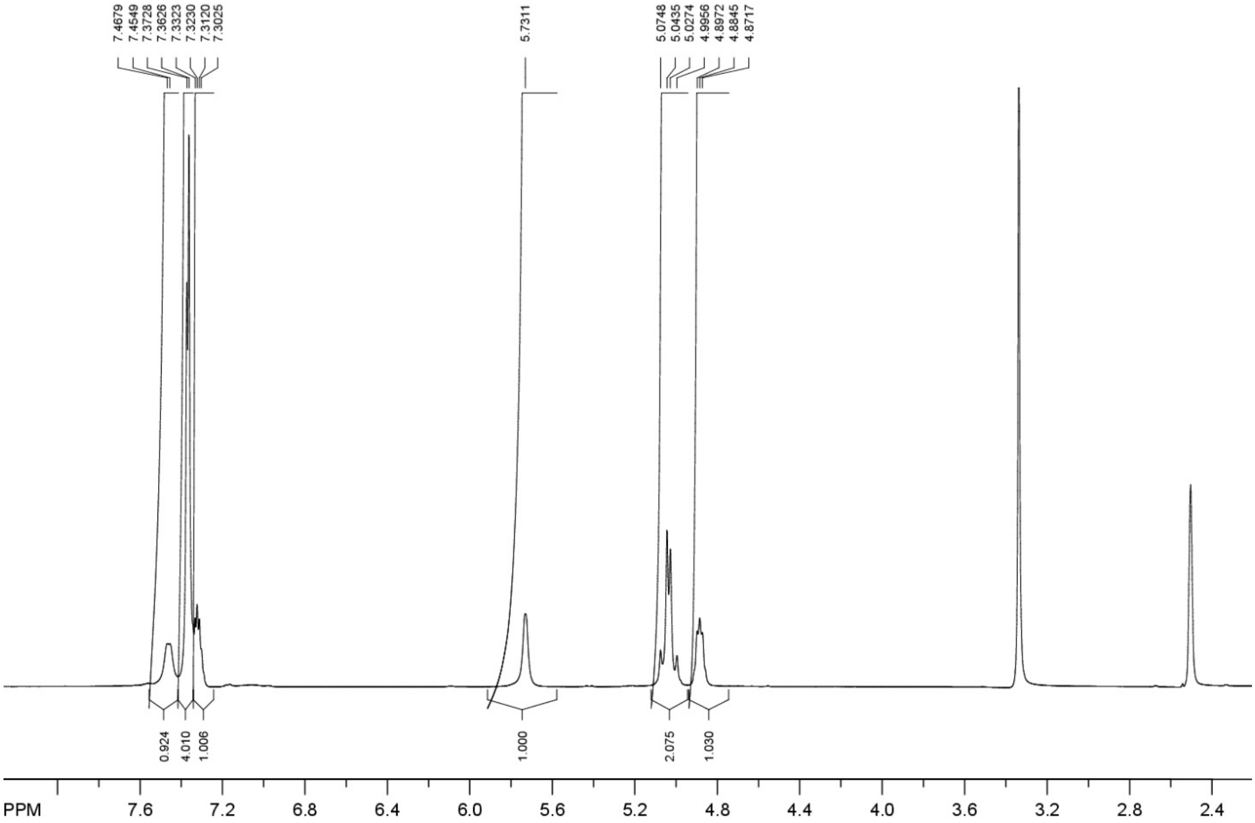

Figure S3. ^{1}H NMR (DMSO-D6) spectrum of compound **3**.

^{13}C (100 MHz) NMR (DMSO-D6, 24°C)

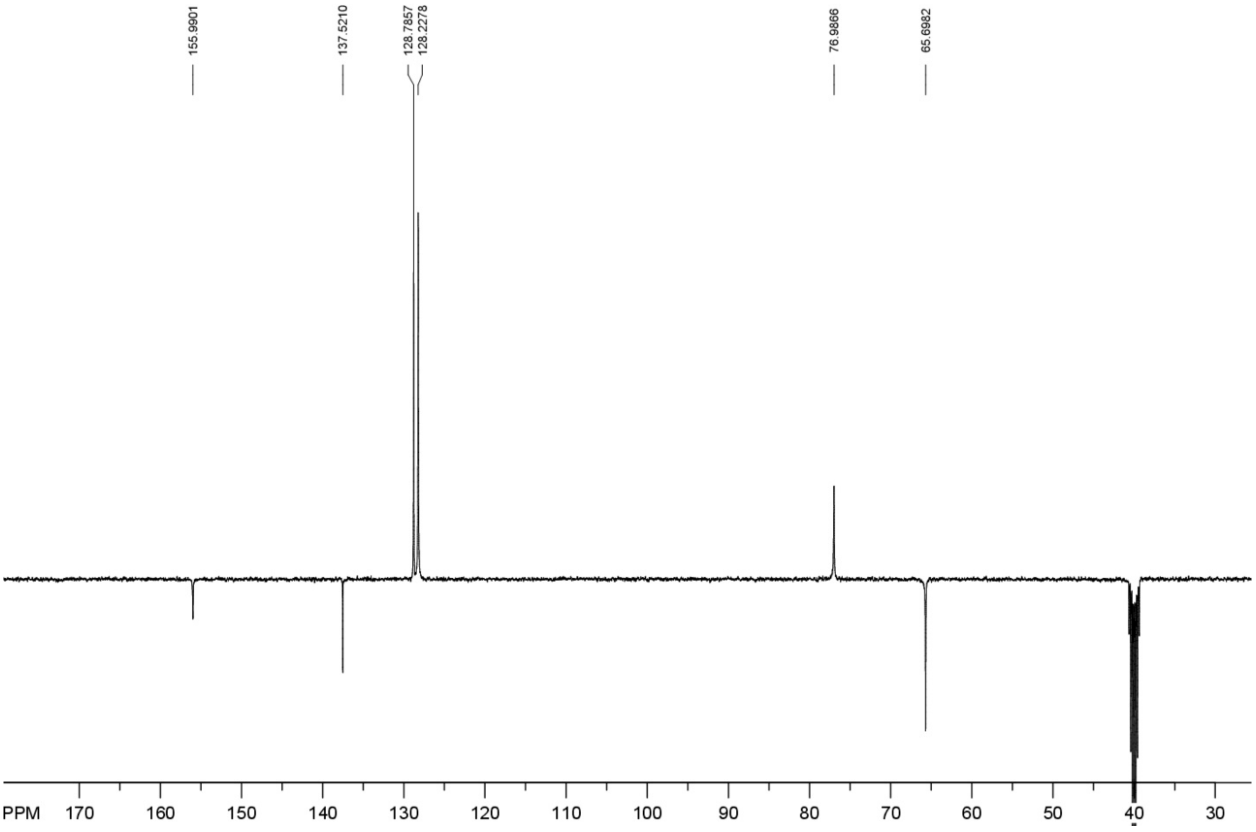

Figure S4. ^{13}C NMR (DMSO-D6) spectrum of compound **3**.

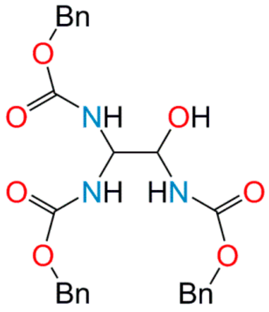

N,N′,N′′-tris(carbobenzoxy)ethanol (**4**)

^1H (400 MHz) NMR (DMSO-D6, 24°C)

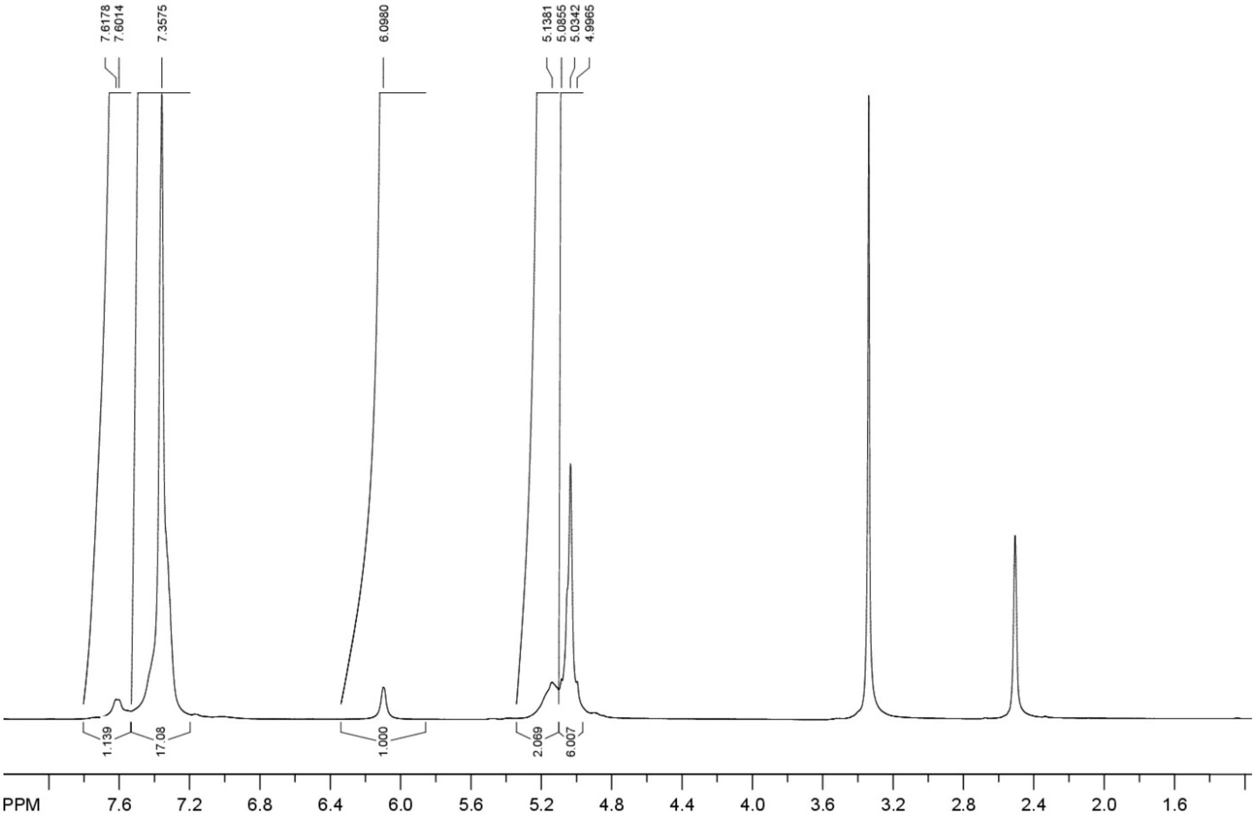

Figure S5. ^1H NMR (DMSO-D6) spectrum of compound **4**.

^{13}C (100 MHz) NMR (DMSO-D6, 24°C)

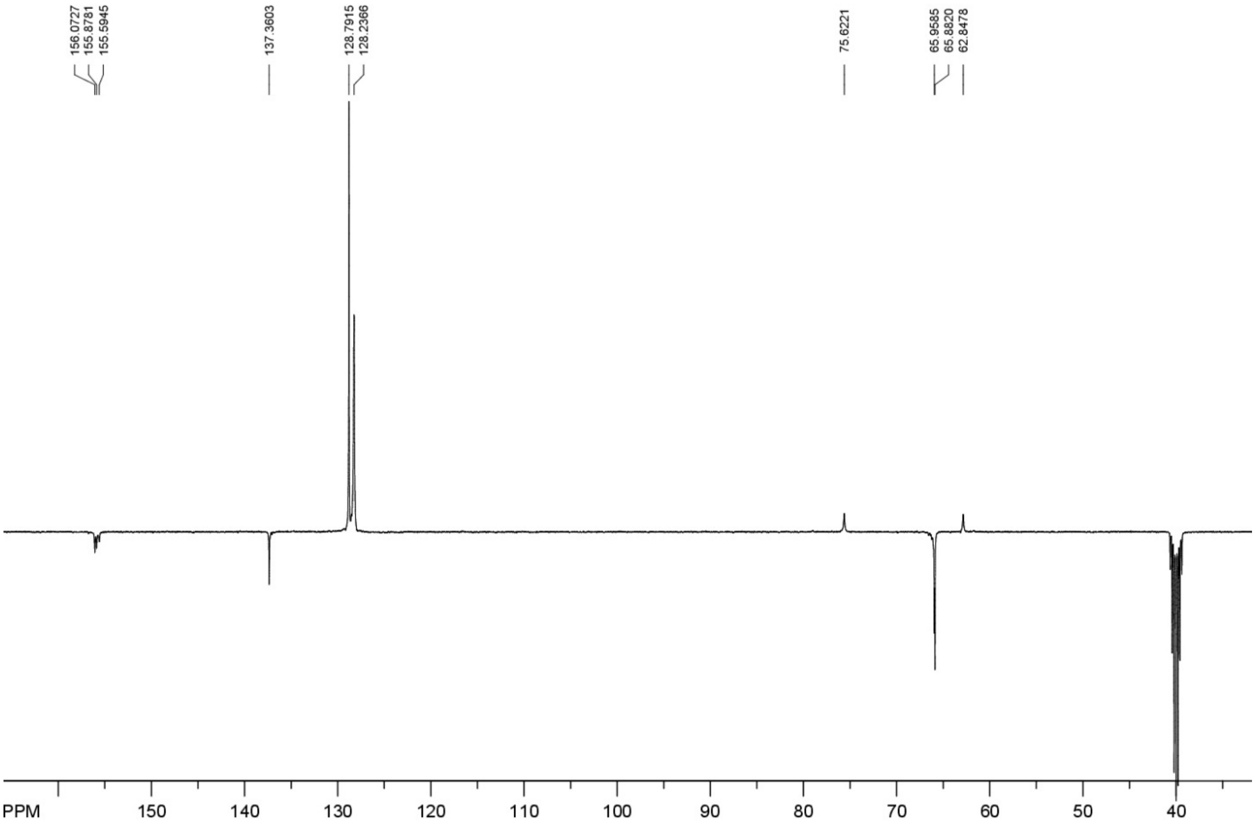

Figure S6. ^{13}C NMR (DMSO-D6) spectrum of compound **4**.

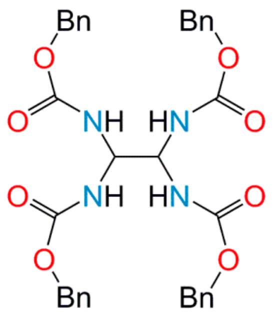

N,N′,N′′,N′′′-tetrakis(carbobenzoxy)ethan (**5**)

^{1}H (400 MHz) NMR (DMSO-D6, 24°C)

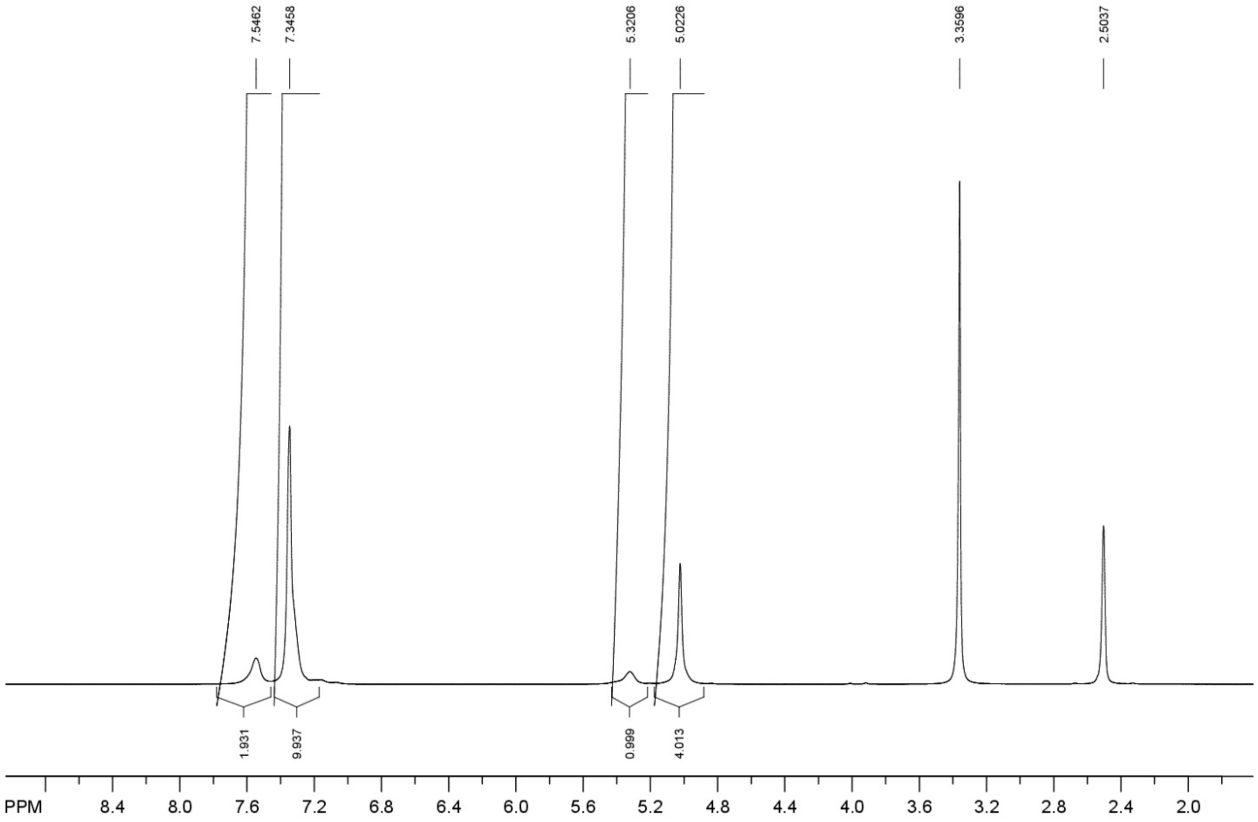

Figure S7. ^{1}H NMR (DMSO-D6) spectrum of compound **5**.

^{13}C (100 MHz) NMR (DMSO-D6, 24°C)

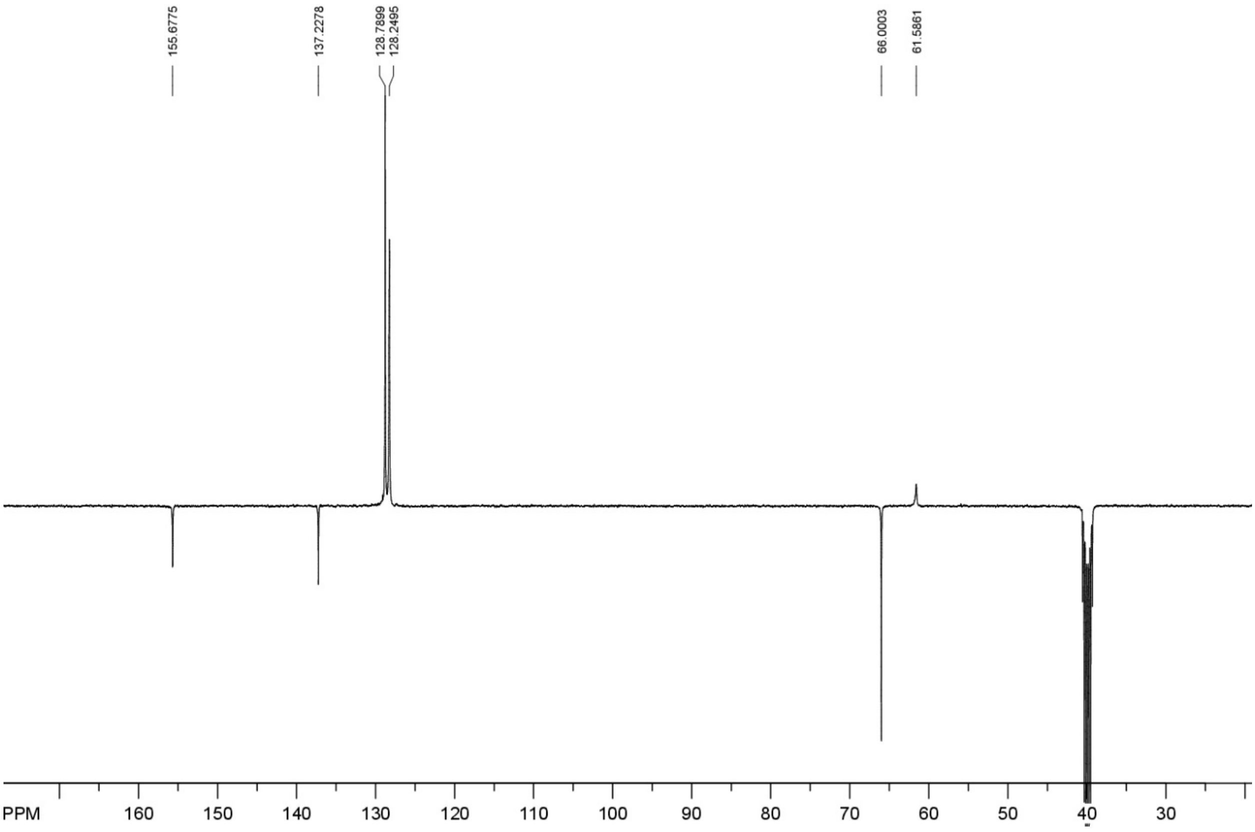

Figure S8. ^{13}C NMR (DMSO-D6) spectrum of compound **5**.

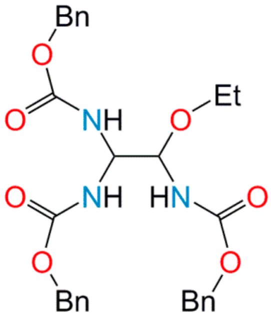

N,N′,N′′-tris(carbobenzoxy)-2-ethoxyethan (**7**)

^{1}H (400 MHz) NMR (DMSO-D6, 24°C); the CH_2 signal overlapped with H_2O.

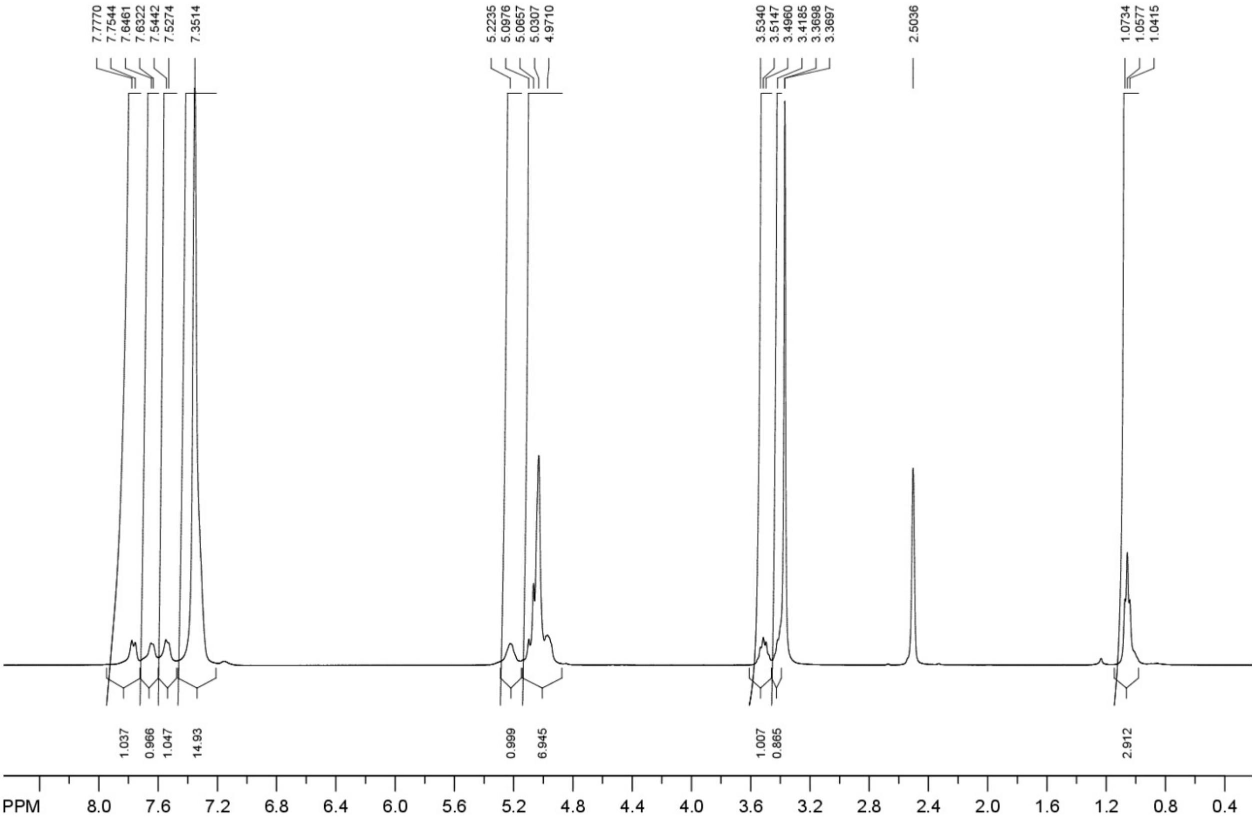

Figure S9. ^{1}H NMR (DMSO-D6) spectrum of compound **7**.

^{1}H (400 MHz) NMR (acetone-D6, 24°C); the compound is much worse soluble in acetone-D6 than in DMSO-D6.

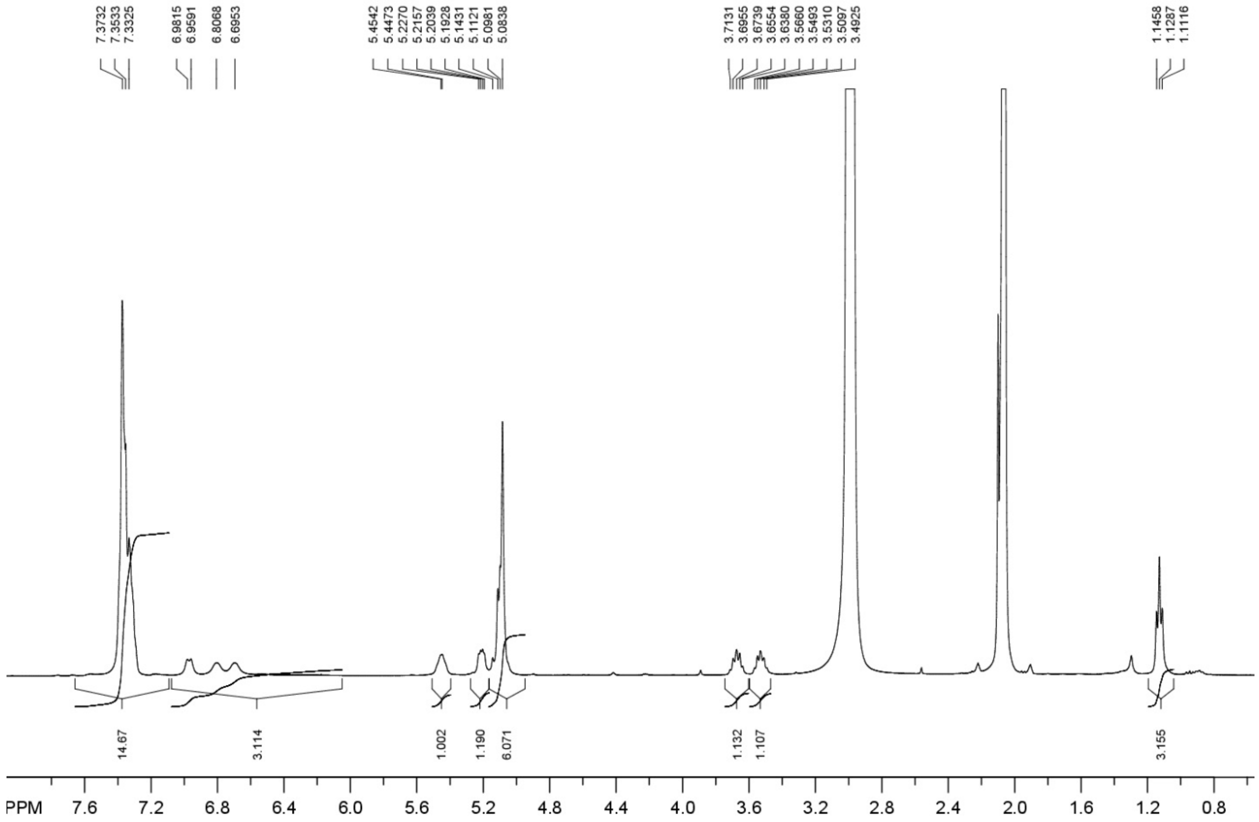

Figure S10. ^{1}H NMR (acetone-D6) spectrum of compound **7**.

^{13}C (100 MHz) NMR (DMSO-D6, 24°C)

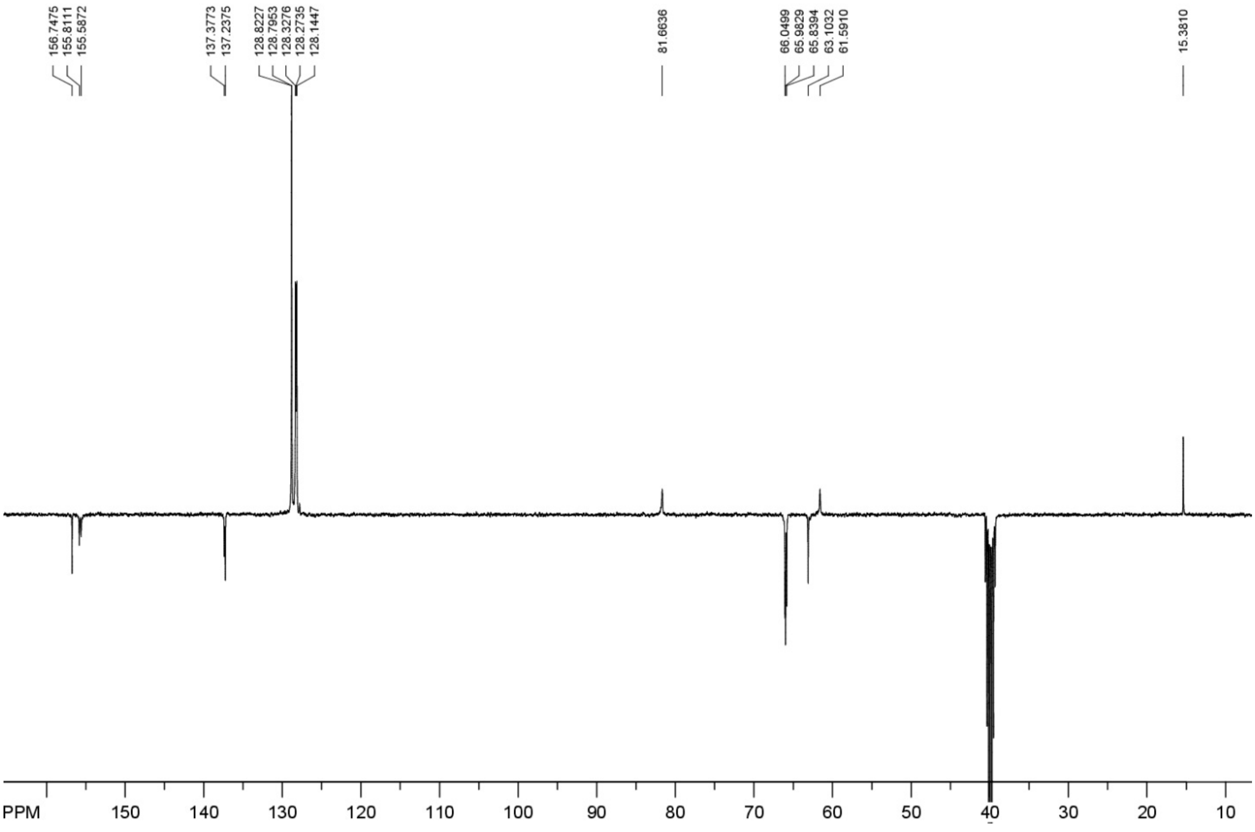

Figure S11. ^{13}C NMR (DMSO-D6) spectrum of compound **7**.

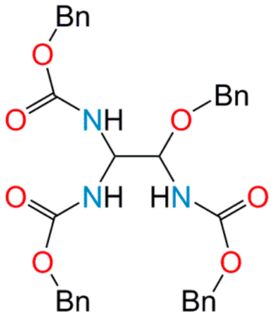

N,N′,N′′-tris(carbobenzoxy)-2-benzoxyethan (**8**)

^{1}H (400 MHz) NMR (DMSO-D6, 24°C)

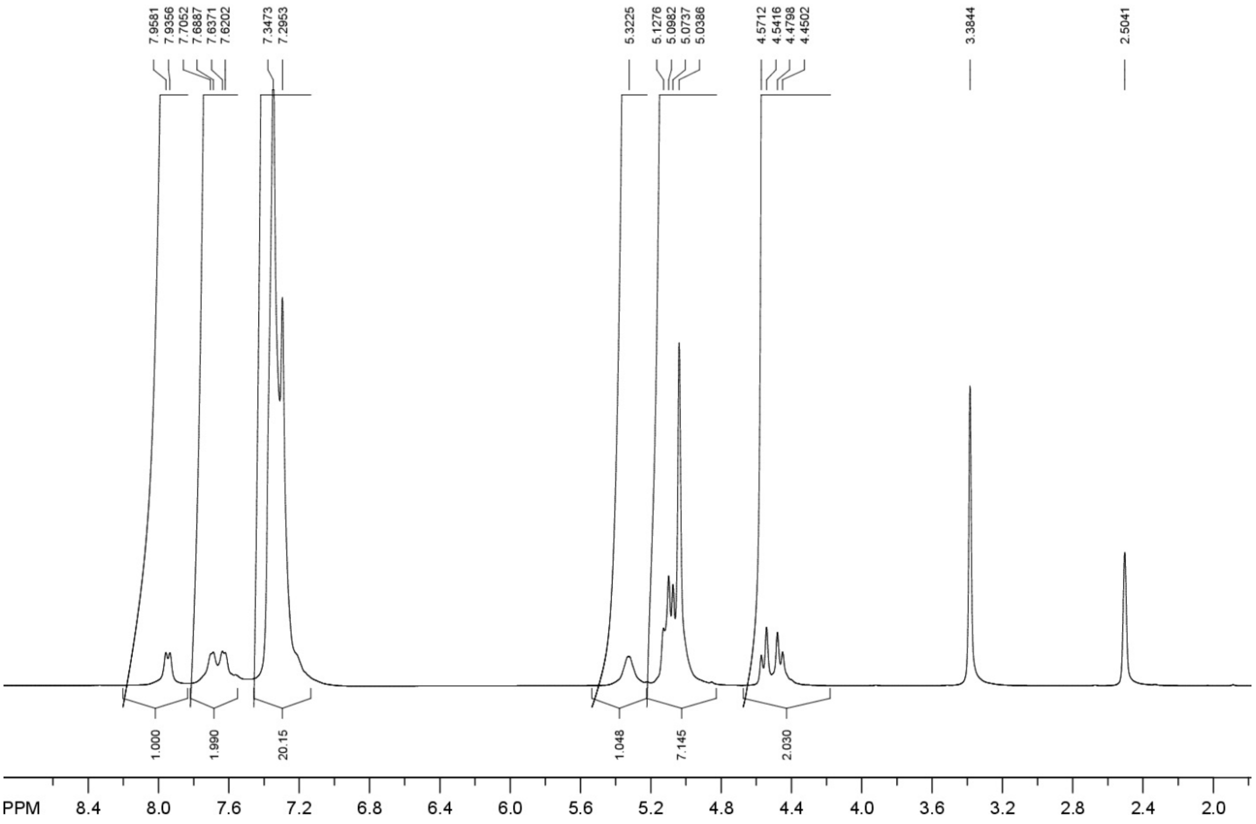

Figure S12. ^{1}H NMR (DMSO-D6) spectrum of compound **8**.

^{13}C (100 MHz) NMR (DMSO-D6, 24°C)

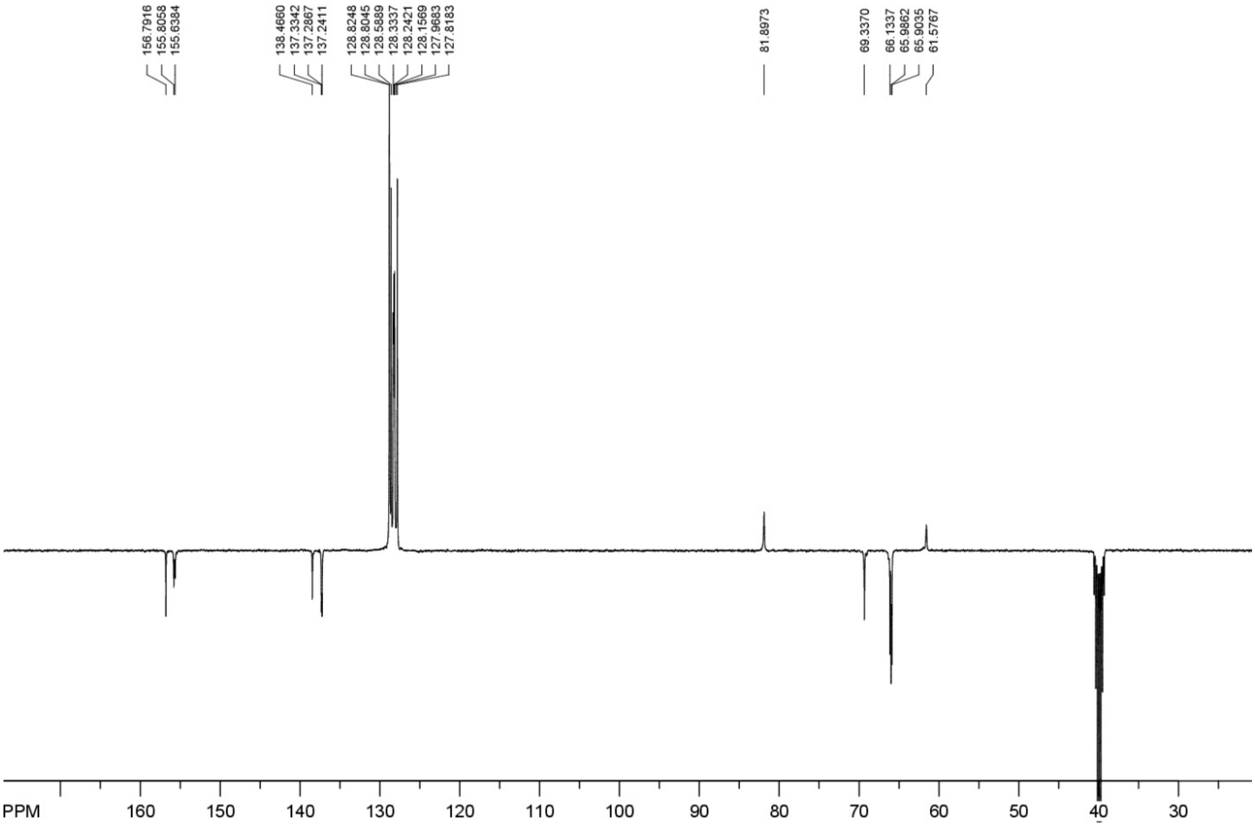

Figure S13. ^{13}C NMR (DMSO-D6) spectrum of compound **8**.
